# Supplementary material for: Carbapenem-Resistant Enterobacteriaceae Bacteremia in Pediatric Patients in Latin America and the Caribbean: A Systematic Review and Meta-Analysis
Source: Antibiotics (Basel). 2024 Nov 22;13(12):1117. doi: 10.3390/antibiotics13121117 (PMC11672711; doi:10.3390/antibiotics13121117)
Supplement: Supplementary file 1 [file antibiotics-13-01117-s001.zip › antibiotics-3227856-supplementary.pdf]

Supplementary material

Supplementary Annex. Search strategy

All databases were searched from January 1 2012 to September 30 2024

PubMed 01/10/2024

Search	Query	Results
#25	#22 AND #23 AND #24	161
#24	(Americas[MeSH Terms:noexp] OR Latin America[Mesh] OR Latin America*[tiab] OR Latinamerica*[tiab] OR Latinoamerica*[tiab] OR Hispanoamerica*[tiab] OR Iberoamerica*[tiab] OR Ibero Americ*[tiab] OR Panamerican*[tiab] OR Central America[Mesh] OR Central America*[tiab] OR Centroamerica*[tiab] OR Mesoamerica*[tiab] OR Meso America*[tiab] OR Middle America*[tiab] OR South America[Mesh] OR South America*[tiab] OR Southamerica*[tiab] OR Sudamerica*[tiab] OR "America del Sur"[tiab] OR Caribbean Region[Mesh] OR Caribbean[tiab] OR Caribe*[tiab] OR West Indies[Mesh] OR West Indi*[tiab] OR Antill*[tiab] OR Indians, South American[Mesh] OR Indians, Central American[Mesh] OR Amerindian*[tiab] OR Indians[tiab] OR American Indian*[tiab] OR Native America*[tiab] OR Patagoni*[tiab] OR Andes[tiab] OR Andean*[tiab] OR Amazon*[tiab] OR Anguilla[ad] OR Anguill*[tiab] OR Anguilla[pl] OR "Antigua and Barbuda"[ad] OR "Antigua and Barbuda"[tiab] OR "Antigua and Barbuda"[pl] OR Argentin*[ad] OR Argentin*[tiab] OR Argentina[pl] OR Bahama*[ad] OR Baham*[tiab] OR Bahama*[pl] OR Bermud*[ad] OR Bermud*[tiab] OR Bermud*[pl] OR Bolivia*[ad] OR Bolivia*[tiab] OR Bolivia[pl] OR Brazil*[ad] OR Brasil*[ad] OR Brazil*[tiab] OR Brasil*[tiab] OR Brazil[pl] OR Cayman*[ad] OR Cayman*[tiab] OR Cayman*[pl] OR Curaçao[ad] OR Curaçao[tiab] OR Curaçao[pl] OR Colombia*[ad] OR Colombia*[tiab] OR Colombia[pl] OR Chile*[ad] OR Chile*[tiab] OR Chile[pl] OR Ecuador*[ad] OR Ecuator*[ad] OR Ecuador*[tiab] OR Ecuador[pl] OR Grenad*[ad] OR Grenad*[tiab] OR Grenad*[pl] OR Guadeloup*[ad] OR Guadeloup*[tiab] OR Guadeloup*[pl] OR Guiana*[ad] OR Guiana*[tiab] OR French Guiana[pl] OR Guyan*[ad] OR Guyan*[tiab] OR Guyana[pl] OR Paraguay*[ad] OR Paraguay*[tiab] OR Paraguay[pl] OR Peru*[ad] OR Peru*[tiab] OR Peru[pl] OR Surinam*[ad] OR Surinam*[tiab] OR Surinam*[pl] OR Uruguay*[ad] OR Uruguay*[tiab] OR Uruguay[pl] OR Venez*[ad] OR Venez*[tiab] OR Venezuela[pl] OR Belize*[ad] OR Belize*[tiab] OR Belize[pl] OR Costa Ric*[ad] OR Costarric*[ad] OR	1,334,482

Search	Query	Results
	Costaric*[ad] OR Costa Ric*[tiab] OR Costarric*[tiab] OR Costaric*[tiab] OR Costa Rica[pl] OR Salvador*[ad] OR Salvador*[tiab] OR El Salvador[pl] OR Guatemal*[ad] OR Guatemal*[tiab] OR Guatemala[pl] OR Hondur*[ad] OR Hondur*[tiab] OR Honduras[pl] OR Martinique[ad] OR Martiniqu*[tiab] OR Martinique[pl] OR Nicaragu*[ad] OR Nicaragu*[tiab] OR Nicaragua[pl] OR Panam*[ad] OR Panam*[tiab] OR Panama[pl] OR Mexico[Mesh] OR Mexic*[ad] OR Mexic*[tiab] OR Mejic*[tiab] OR Mexico[pl] OR Montserrat[ad] OR Montserrat*[tiab] OR Montserrat[pl] OR Baham*[ad] OR Baham*[tiab] OR Bahamas[pl] OR Cuba*[ad] OR Cuba*[tiab] OR Cuba[pl] OR Dominic*[ad] OR Dominic*[tiab] OR Dominican Republic[pl] OR Haiti*[ad] OR Haiti*[tiab] OR Haiti[pl] OR Jamaic*[ad] OR Jamaic*[tiab] OR Jamaica[pl] OR Puerto Rico[Mesh] OR Puerto Ric*[tiab] OR Puertorric*[tiab] OR Puertoric*[tiab] OR Saint Kitts[ad] OR Saint Kitts[tiab] OR Saint Kitts[pl] OR "Trinidad and Tobago"[tiab] OR "Trinidad and Tobago"[ad] OR "Trinidad and Tobago"[pl])	
#23	(Infant[Mesh] OR Infant*[tiab] OR Neonat*[tiab] OR Newborn*[tiab] OR Pediatric*[tiab] OR Paediatric*[tiab] OR Child*[tiab] OR Babies[tiab] OR Adolescent[Mesh] OR Adolescen*[tiab] OR Teenage*[tiab] OR Teens[tiab] OR Juvenil*[tiab] OR Youth*[tiab] OR Toddler*[tiab])	4,362,720
#22	#18 OR #19 OR #20 OR #21	14,613
#21	Carbapenemase-Producing Enterobacter*[tiab]	1,449
#20	Carbapenem Resist*[tiab]	8,761
#19	Carbapenem-Resistant Enterobacteriaceae[Mesh]	1,280
#18	#16 AND #17	10,943
#17	Resist*[tiab]	1,222,060
#16	#3 AND #15	13,368
#15	#4 OR #5 OR #6 OR #7 OR #8 OR #9 OR #10 OR #11 OR #12 OR #13 OR #14	547,499
#14	Providencia[tiab]	1,730
#13	Shigella[tiab]	15,677

Search	Query	Results
#12	Klebsiella[tiab]	38,191
#11	Escherichia*[tiab]	296,624
#10	Leclercia[tiab]	192
#9	Ewingella[tiab]	68
#8	Paracolobact*[tiab]	216
#7	Sodalis[tiab]	238
#6	Coliform Bacilli[tiab]	93
#5	Enterobacteria*[tiab]	27,811
#4	Enterobacteriaceae[Mesh]	427,262
#3	#1 OR #2	26,747
#2	Carbapenem*[tiab]	20,536
#1	Carbapenems[Mesh]	14,078

EMBase 1/10/2024

Embase <1974 to 2024 September 30>

1	exp carbapenem derivative/	11119
2	Carbapenem*.ti,ab.	27762
3	1 or 2	32019
4	exp Enterobacteriaceae/	551927
5	exp Enterobacteriaceae infection/	58732
6	Enterobacteria*.ti,ab.	32421
7	(Coliform adj1 Bacilli).ti,ab.	60
8	Sodalis.ti,ab.	228
9	Paracolobact*.ti,ab.	1
10	Ewingella.ti,ab.	71
11	Leclercia.ti,ab.	216
12	Escherichia*.ti,ab.	302274
13	Klebsiella.ti,ab.	50518
14	Shigella.ti,ab.	15224
15	Providencia.ti,ab.	1931
16	or/4-15	643529
17	3 and 16	18691

18 Resist*.ti,ab. 1493841
19 17 and 18 15519
20 exp carbapenem-resistant Enterobacteriaceae/ 4075
21 (Carbapenem adj1 Resist*).ti,ab. 11114
22 (Carbapenemase-Producing adj3 Enterobacter*).ti,ab. 1791
23 20 or 21 or 22 13229
24 19 or 23 20150
25 exp infant/ 1089219
26 Infant*.ti,ab. 511609
27 Neonat*.ti,ab. 387106
28 Newborn*.ti,ab. 208479
29 Pediatric*.ti,ab. 533112
30 Paediatric*.ti,ab. 130201
31 Child*.ti,ab. 1969588
32 Babies.ti,ab. 57593
33 exp adolescent/ 1698427
34 Adolescen*.ti,ab. 426356
35 Teenage*.ti,ab. 32271
36 Teens.ti,ab. 10087
37 Juvenil*.ti,ab. 109500
38 Youth*.ti,ab. 110638
39 Toddler*.ti,ab. 17282
40 or/25-394382558
41 exp "South and Central America"/ 274950
42 exp South America/ or exp Central America/ or (Latin adj1 America*).ti,ab. or Latinamerica*.ti,ab. or Latinoamerica*.ti,ab. or Hispanoamerica.ti,ab. or Iberoamerica*.ti,ab. or (Ibero adj1 Americ*).ti,ab. or Panamerica*.ti,ab. or (South adj1 America*).ti,ab. or Southamerica*.ti,ab. or Sudamerica*.ti,ab. or (America adj1 Sur).ti,ab. or (Central adj1 America*).ti,ab. or Centroamerica*.ti,ab. or Mesoamerica*.ti,ab. or (Meso adj1 America*).ti,ab. or (Middle adj1 America*).ti,ab. or exp Caribbean Islands/ or Caribbean*.ti,ab. or Caribe*.ti,ab. or (West adj1 Indi*).ti,ab. or Antill*.ti,ab. or exp American indian/ or Amerindian*.ti,ab. or Indians.ti,ab. or (Native adj1 America*).ti,ab. or Patagoni*.ti,ab. or Andes.ti,ab. or Andean*.ti,ab. or Amazon*.ti,ab. or exp Argentina/ or Argentin*.ti,ab. or exp Bolivia/ or Bolivia*.ti,ab. or exp Brazil/ or Brazil*.ti,ab. or Brasil*.ti,ab. or exp Colombia/ or Colombia*.ti,ab. or exp Chile/ or Chile*.ti,ab. or exp Ecuador/ or Ecuador*.ti,ab. or exp French Guiana/ or Guiana*.ti,ab. or exp Guyana/ or Guyan*.ti,ab. or exp Paraguay/ or Paraguay*.ti,ab. or exp Peru/ or Peru*.ti,ab. or exp Suriname/ or Surinam*.ti,ab. or exp Uruguay/ or Uruguay*.ti,ab. or exp Venezuela/ or Venez*.ti,ab. or exp Belize/ or Beliz*.ti,ab. or exp Costa Rica/ or (Costa adj1 Rica).ti,ab. or Costarric*.ti,ab. or Costaric*.ti,ab. or exp El salvador/ or Salvador*.ti,ab. or exp Guatemala/ or Guatemal*.ti,ab. or exp Honduras/ or Hondur*.ti,ab. or exp Nicaragua/ or Nicaragu*.ti,ab. or exp Panama/ or Panam*.ti,ab. or exp Mexico/ or Mexic*.ti,ab. or exp Cuba/ or Cuba*.ti,ab. or exp Dominican Republic/ or Dominica*.ti,ab. or exp Haiti/ or Haiti*.ti,ab. or exp Jamaica/ or Jamaic*.ti,ab. or exp Puerto Rico/ or (Puerto adj1 Ric*).ti,ab. or Puertoric*.ti,ab. or Puertorric*.ti,ab.573777
43 41 or 42 576381
44 24 and 40 and 43 127

LILACS 1/10/2024

Database :	LILACS
Search on :	((MH Carbapenems OR Carbapenem$) AND (MH Enterobacteriaceae OR Enterobacteria$ OR Coliform OR Sodalis OR Paracolobact$ OR Ewingella$ OR Leclercia$ OR Escherichia$ OR Klebsiella$ OR Shigella$ OR Shigela$ OR Providencia$) AND (Resist$)) OR (MH Carbapenem-Resistant Enterobacteriaceae OR ((Carbapenem$) AND (Resist$ OR Producing OR Productor$ OR Produtor$))) AND (MH Infant OR Infant$ OR Neonat$ OR Newborn$ OR Pediatric$ OR Paediatric$ OR Child$ OR Niño$ OR Menino$ OR Babies OR Bebe OR Bebés OR Criança$ OR MH Adolescent OR Adolescen$ OR Teenage$ OR Teens OR Juvenil$ OR Youth$ OR Toddler$) [Words]
References found :	395 [refine]

CINAHL 1/10/2024

#	Query	Results
S43	S23 AND S39 AND S42	19
S42	S40 OR S41	134,560
S41	AB (Latin N1 America*) OR Latinamerica* OR Latinoamerica* OR Latin* OR Hispanic Americans OR Iberoamerica* OR (Ibero N1 Americ*) OR Panamerican* OR (Central N1 America*) OR Centroamerica* OR Mesoamerica* OR (Meso N1 America*) OR (Middle N1 America*) OR (South N1 America*) OR Southamerica* OR Sudamerica* OR (America N1 Sur) OR Caribbean OR Caribe* OR (West N1 Indi*) OR Antill* OR Amerindian* OR Indians OR (American N1 Indian*) OR (Native N1 America*) OR Patagoni* OR Andes OR Andean* OR Amazon* OR Argentin* OR Bolivia* OR Brazil* OR Brasil* Colombia* OR Colombia* OR Colombia OR Chile* OR Ecuador* OR Guiana* OR Guyan* OR Guyan* OR Paraguay* OR Paraguay* OR Peru* OR Surinam* OR Surinam* OR Uruguay* OR Venez* OR Belize* OR (Costa N1 Ric*) OR Costarric* OR Costaric* OR Costa Ric* OR Costarric* OR Salvador* OR Salvador* OR Guatemal* OR Guatemal* OR	106,424

	Guatemala OR Hondur* OR Nicaragu* OR Panam* OR Mexic* OR Cuba* OR Dominic* OR Dominic* OR Haiti* OR Jamaic* OR (Puerto N1 Ric*) OR Puertorric* OR Puertoric*	
S40	TI (Latin N1 America*) OR Latinamerica* OR Latinoamerica* OR Latin* OR Hispanic Americans OR Iberoamerica* OR (Ibero N1 Americ*) OR Panamerican* OR (Central N1 America*) OR Centroamerica* OR Mesoamerica* OR (Meso N1 America*) OR (Middle N1 America*) OR (South N1 America*) OR Southamerica* OR Sudamerica* OR (America N1 Sur) OR Caribbean OR Caribe* OR (West N1 Indi*) OR Antill* OR Amerindian* OR Indians OR (American N1 Indian*) OR (Native N1 America*) OR Patagoni* OR Andes OR Andean* OR Amazon* OR Argentin* OR Bolivia* OR Brazil* OR Brasil* Colombia* OR Colombia* OR Colombia OR Chile* OR Ecuador* OR Guiana* OR Guyan* OR Guyan* OR Paraguay* OR Paraguay* OR Peru* OR Surinam* OR Surinam* OR Uruguay* OR Venez* OR Belize* OR (Costa N1 Ric*) OR Costarric* OR Costaric* OR Costa Ric* OR Costarric* OR Salvador* OR Salvador* OR Guatemal* OR Guatemal* OR Guatemala OR Hondur* OR Nicaragu* OR Panam* OR Mexic* OR Cuba* OR Dominic* OR Dominic* OR Haiti* OR Jamaic* OR (Puerto N1 Ric*) OR Puertorric* OR Puertoric*	70,698
S39	S24 OR S25 OR S26 OR S27 OR S28 OR S29 OR S30 OR S31 OR S32 OR S33 OR S34 OR S35 OR S36 OR S37 OR S38	1,309,185
S38	TI Toddler* OR AB Toddler*	7,856
S37	TI Youth* OR AB Youth*	60,353
S36	TI Juvenil* OR AB Juvenil*	11,883
S35	TI Teens OR AB Teens	10,187
S34	TI Teenage* OR AB Teenage*	11,914
S33	TI Adolescen* OR AB Adolescen*	160,185

S32	(MH "Adolescence+")	589,766
S31	TI Babies OR AB Babies	38,156
S30	TI Child* OR AB Child*	572,115
S29	TI Paediatric* OR AB Paediatric*	29,949
S28	TI Pediatric* OR AB Pediatric*	137,352
S27	TI Newborn* OR AB Newborn*	36,349
S26	TI Neonat* OR AB Neonat*	79,549
S25	TI Infant* OR AB Infant*	123,726
S24	(MH "Infant+")	287,242
S23	S18 OR S22	2,482
S22	S19 OR S20 OR S21	1,975
S21	TI (Carbapenemase-Producing N1 Enterobacter*) OR AB (Carbapenemase-Producing N1 Enterobacter*)	346
S20	TI (Carbapenem N1 Resist*) OR AB (Carbapenem N1 Resist*)	1,609
S19	(MH "Carbapenem-Resistant Enterobacteriaceae")	247
S18	S16 AND S17	1,766
S17	TI Resist* OR AB Resist*	144,179
S16	S3 AND S15	2,264
S15	S4 OR S5 OR S6 OR S7 OR S8 OR S9 OR S10 OR S11 OR S12 OR S13 OR S14	18,871
S14	TI Providencia OR AB Providencia	100
S13	TI Shigella OR AB Shigella	914
S12	TI Klebsiella OR AB Klebsiella	4,028
S11	TI Escherichia* OR AB Escherichia*	9,123
S10	TI Leclercia OR AB Leclercia	15
S9	TI Ewingella OR AB Ewingella	3
S8	TI Paracolobact* OR AB Paracolobact*	0

S7	TI Sodalis OR AB Sodalis	4
S6	TI (Coliform N1 Bacilli) OR AB (Coliform N1 Bacilli)	2
S5	TI Enterobacteria* OR AB Enterobacteria*	2,582
S4	(MH "Enterobacteriaceae+")	11,543
S3	S1 OR S2	4,187
S2	TI Carbapenem* OR AB Carbapenem*	3,304
S1	(MH "Carbapenems+")	2,027

SciELO 1/10/2024

((Carbapenem$) AND (Enterobacteria$ OR Coliform OR Sodalis OR Paracolobact$ OR Ewingella$ OR Leclercia$ OR Escherichia$ OR Klebsiella$ OR Shigella$ OR Shigela$ OR Providencia$) AND (Resist$)) OR (((Carbapenem$) AND (Resist$ OR Producing OR Productor$ OR Produtor$))) AND (Infant$ OR Neonat$ OR Newborn$ OR Pediatric$ OR Paediatric$ OR Child$ OR Niño$ OR Menino$ OR Babies OR Bebe OR Bebés OR Criança$ OR Adolescen$ OR Teenage$ OR Teens OR Juvenil$ OR Youth$ OR Toddler$) Resultados: **42**

Supplementary Table S1. PRISMA 2020 checklist

PRISMA 2020 Checklist

Section and Topic	Item #	Checklist item	Location where item is reported
**TITLE**			
Title	1	Identify the report as a systematic review.	1
**ABSTRACT**			
Abstract	2	See the PRISMA 2020 for Abstracts checklist.	1-2
**INTRODUCTION**			
Rationale	3	Describe the rationale for the review in the context of existing knowledge.	3
Objectives	4	Provide an explicit statement of the objective(s) or question(s) the review addresses.	3
**METHODS**			
Eligibility criteria	5	Specify the inclusion and exclusion criteria for the review and how studies were grouped for the syntheses.	3-4
Information sources	6	Specify all databases, registers, websites, organisations, reference lists and other sources searched or consulted to identify studies. Specify the date when each source was last searched or consulted.	3
Search strategy	7	Present the full search strategies for all databases, registers and websites, including any filters and limits used.	Supplementary material (pages 1-8)

Selection process	8	Specify the methods used to decide whether a study met the inclusion criteria of the review, including how many reviewers screened each record and each report retrieved, whether they worked independently, and if applicable, details of automation tools used in the process.	4
Data collection process	9	Specify the methods used to collect data from reports, including how many reviewers collected data from each report, whether they worked independently, any processes for obtaining or confirming data from study investigators, and if applicable, details of automation tools used in the process.	4
Data items	10a	List and define all outcomes for which data were sought. Specify whether all results that were compatible with each outcome domain in each study were sought (e.g. for all measures, time points, analyses), and if not, the methods used to decide which results to collect.	3-4
	10b	List and define all other variables for which data were sought (e.g. participant and intervention characteristics, funding sources). Describe any assumptions made about any missing or unclear information.	3-4
Study risk of bias assessment	11	Specify the methods used to assess risk of bias in the included studies, including details of the tool(s) used, how many reviewers assessed each study and whether they worked independently, and if applicable, details of automation tools used in the process.	4
Effect measures	12	Specify for each outcome the effect measure(s) (e.g. risk ratio, mean difference) used in the synthesis or presentation of results.	4
Synthesis methods	13a	Describe the processes used to decide which studies were eligible for each synthesis (e.g. tabulating the study intervention characteristics and comparing against the planned groups for each synthesis (item #5)).	4
	13b	Describe any methods required to prepare the data for presentation or synthesis, such as handling of missing summary statistics, or data conversions.	4
	13c	Describe any methods used to tabulate or visually display results of individual studies and syntheses.	4
	13d	Describe any methods used to synthesize results and provide a rationale for the choice(s). If meta-analysis was performed, describe the model(s), method(s) to identify the presence and extent of statistical heterogeneity, and software package(s) used.	4

	13e	Describe any methods used to explore possible causes of heterogeneity among study results (e.g. subgroup analysis, meta-regression).	4
	13f	Describe any sensitivity analyses conducted to assess robustness of the synthesized results.	4
Reporting bias assessment	14	Describe any methods used to assess risk of bias due to missing results in a synthesis (arising from reporting biases).	4
Certainty assessment	15	Describe any methods used to assess certainty (or confidence) in the body of evidence for an outcome.	4

Section and Topic	Item #	Checklist item	Location where item is reported
**RESULTS**			
Study selection	16a	Describe the results of the search and selection process, from the number of records identified in the search to the number of studies included in the review, ideally using a flow diagram.	Figure 1
	16b	Cite studies that might appear to meet the inclusion criteria, but which were excluded, and explain why they were excluded.	Supplementary table S2
Study characteristics	17	Cite each included study and present its characteristics.	Table 1
Risk of bias in studies	18	Present assessments of risk of bias for each included study.	6, supplementary tables S3 and S4

Results of individual studies	19	For all outcomes, present, for each study: (a) summary statistics for each group (where appropriate) and (b) an effect estimate and its precision (e.g. confidence/credible interval), ideally using structured tables or plots.	5,14
Results of syntheses	20a	For each synthesis, briefly summarise the characteristics and risk of bias among contributing studies.	NA
	20b	Present results of all statistical syntheses conducted. If meta-analysis was done, present for each the summary estimate and its precision (e.g. confidence/credible interval) and measures of statistical heterogeneity. If comparing groups, describe the direction of the effect.	Supplementary table S3
	20c	Present results of all investigations of possible causes of heterogeneity among study results.	7,8
	20d	Present results of all sensitivity analyses conducted to assess the robustness of the synthesized results.	NA
Reporting biases	21	Present assessments of risk of bias due to missing results (arising from reporting biases) for each synthesis assessed.	NA
Certainty of evidence	22	Present assessments of certainty (or confidence) in the body of evidence for each outcome assessed.	NA
**DISCUSSION**			
Discussion	23a	Provide a general interpretation of the results in the context of other evidence.	6,7
	23b	Discuss any limitations of the evidence included in the review.	7,8
	23c	Discuss any limitations of the review processes used.	NA
	23d	Discuss implications of the results for practice, policy, and future research.	8
**OTHER INFORMATION**			
Registration and	24a	Provide registration information for the review, including register name and registration number, or state that the review was not registered.	3

protocol	24b	Indicate where the review protocol can be accessed, or state that a protocol was not prepared.	3
	24c	Describe and explain any amendments to information provided at registration or in the protocol.	NA
Support	25	Describe sources of financial or non-financial support for the review, and the role of the funders or sponsors in the review.	8
Competing interests	26	Declare any competing interests of review authors.	NA
Availability of data, code and other materials	27	Report which of the following are publicly available and where they can be found: template data collection forms; data extracted from included studies; data used for all analyses; analytic code; any other materials used in the review.	NA

From: Page MJ, McKenzie JE, Bossuyt PM, Boutron I, Hoffmann TC, Mulrow CD, et al. The PRISMA 2020 statement: an updated guideline for reporting systematic reviews. BMJ 2021;372:n71. doi: 10.1136/bmj.n71

For more information, visit: http://www.prisma-statement.org/

Supplementary Table S2. Studies excluded in full text and reason for exclusion

Author and year of publication	Reason for exclusion
Akturk, 2016	Wrong setting
Bilderberg, 2015	Wrong study population
Canales Fuentes, 2016	Wrong outcome
Costa, 2014	Wrong outcome
Costa, 2015	Wrong outcome
Costa, 2020	Wrong study population
De Luna, 2019	Wrong outcome
Garzón, 2020	Wrong outcome
Graff, 2020	Duplicate
Oliva, 2021	Wrong outcome
Quiñonez Pérez, 2020	Wrong outcome
Rada, 2020	Wrong outcome
Rojas, 2018	Wrong study population
Rosenthal, 2021	Wrong study population
Stewardson, 2019	Wrong study population
Uc- Cachon, 2019	Wrong study population
Vanegas, 2016	Wrong outcome
Villalobos, 2014	Wrong study population

Supplementary Table S3. Risk of Bias Assessment for Case Series studies:

Author and year of publication	Evaluation *									
	1	2	3	4	5	6	7	8	9	Final
**Alvares 2019** [21]	**Yes**	**Yes**	**Yes**	**Yes**	**Yes**	**Yes**	**Yes**	**Yes**	**Yes**	**Good**
**Aquino Andrade 2018** [28]	**Yes**	**Yes**	**Yes**	**Yes**	**Yes**	**Yes**	**Yes**	**No**	**Yes**	**Good**
**de Oliveira 2015** [22]	**Yes**	**Yes**	**CD**	**Yes**	**Yes**	**Yes**	**Yes**	**Yes**	**Yes**	**Good**
**Higashino 2018** [23]	**Yes**	**Yes**	**Yes**	**Yes**	**Yes**	**Yes**	**Yes**	**Yes**	**Yes**	**Good**
**Marquez Herrera 2016** [25]	**Yes**	**Yes**	**CD**	**Yes**	**Yes**	**Yes**	**Yes**	**Yes**	**Yes**	**Good**
**Perez 2020** [26]	**Yes**	**Yes**	**CD**	**Yes**	**Yes**	**Yes**	**Yes**	**Yes**	**Yes**	**Good**
**Reyes Chacon 2021** [27]	**Yes**	**Yes**	**CD**	**Yes**	**Yes**	**Yes**	**Yes**	**Yes**	**Yes**	**Good**
**Touchet 2021** [30]	**Yes**	**Yes**	**Yes**	**NA**	**Yes**	**Yes**	**Yes**	**No**	**Yes**	**Fair**
**López Cubillos** [32]	**Yes**	**Yes**	**Yes**	**Yes**	**Yes**	**Yes**	**Yes**	**Yes**	**Yes**	**Good**

* **NA**: Not applicable, **CD**: Cannot be determined

1. Was the study question or objective clearly specified?
2. Was the study population clearly and fully described, including case definition?
3. Were cases consecutive?
4. Were subjects comparable?
5. Was exposure clearly described?
6. Were measures of results clearly defined, valid, reliable and consistently implemented for all study participants?
7. Was the length of follow-up appropriate?
8. Were statistical methods properly described?
9. Were results properly described?

Supplementary Table S4. Risk of Bias Assessment for Case- control studies

Author and year of publication	Evaluation *												
	1	2	3	4	5	6	7	8	9	10	11	12	Final
**Ruvinsky 2022** [20]	**Yes**	**Yes**	**No**	**Yes**	**Yes**	**Yes**	**No**	**Yes**	**Yes**	**Yes**	**No**	**Yes**	**Good**

* **NA**: Not applicable, **CD**: Cannot be determined

1. Was the research question or objective in this paper clearly stated and appropriate?
2. Was the study population clearly specified and defined?
3. Did the authors include a sample size justification?
4. Were controls selected or recruited from the same or similar population that gave rise to the cases (including the same timeframe)?
5. Were the definitions, inclusion and exclusion criteria, algorithms or processes used to identify or select cases and controls valid, reliable, and implemented consistently across all study participants?
6. Were the cases clearly defined and differentiated from controls?
7. If less than 100 percent of eligible cases and/or controls were selected for the study, were the cases and/or controls randomly selected from those eligible?
8. Was there use of concurrent controls?
9. Were the investigators able to confirm that the exposure/risk occurred prior to the development of the condition or event that defined a participant as a case?
10. Were the measures of exposure/risk clearly defined, valid, reliable, and implemented consistently (including the same time period) across all study participants?
11. Were the assessors of exposure/risk blinded to the case or control status of participants?
12. Were key potential confounding variables measured and adjusted statistically in the analyses? If matching was used, did the investigators account for matching during study analysis?
